# Cardiovascular causes of sudden unexpected death in children and adolescents (0–17 years): A nationwide autopsy study in the Netherlands

**DOI:** 10.1007/s12471-018-1152-y

**Published:** 2018-09-03

**Authors:** A. Vos, A. C. van der Wal, A. H. Teeuw, J. Bras, A. Vink, P. G. J. Nikkels, Dutch NODO group

**Affiliations:** 1 Department of Pathology, University Medical Center, Utrecht, The Netherlands; 2 Department of Pathology, Academic Medical Center, Amsterdam, The Netherlands; 3 Department of Paediatrics, Academic Medical Center, Amsterdam, The Netherlands

**Keywords:** Autopsy, Sudden death, Cardiovascular diseases, Cardiomyopathies, Myocarditis

## Abstract

**Background:**

Little is known about the causes of unexpected death in minors (0–17 years). In young adults an important cause is cardiovascular disease, with primary arrhythmogenic disorders, atherosclerotic events, cardiomyopathies and myocarditis as main contributors. The aim of this autopsy study was to determine the contribution of cardiovascular disease to unexpected death in minors.

**Methods and results:**

In the Netherlands, systematic investigation of all cases of unexplained death in minors was compulsory in a nationwide governmental project during a 15-month period. Autopsies were performed according to a standardised protocol (autopsy rate 85%). A cardiovascular cause of death was found in 13/56 cases (23%). In the group <1 year, the main cardiovascular causes were various congenital defects (*n* = 3) and myocarditis (*n* = 2). In the 1–9 year group, no cardiovascular causes were found. In the 10–14 year group, hypertrophic cardiomyopathy (*n* = 1) and ruptured ascending aortic aneurysm (*n* = 1) were among the observed cardiovascular causes. In 14/56 (25%) cases autopsy revealed no structural abnormalities that could explain the sudden death, mostly in the group <1 year.

**Conclusion:**

This national cohort with a high autopsy rate reveals a high incidence (23%) of cardiovascular diseases as the pathological substrate of sudden unexpected death in children. Another high percentage of minors (25%) showed no structural abnormalities, with the possibility of a genetic arrhythmia. These findings underline the importance of systematic autopsy in sudden death in minors, with implications for cardiogenetic screening of relatives.

**Electronic supplementary material:**

The online version of this article (10.1007/s12471-018-1152-y) contains supplementary material, which is available to authorized users.

## What’s new

- Cardiovascular diseases are an important cause of sudden unexpected death in children (age 0–17; 23%).
- In 25% of sudden unexpected death cases in children (0–17 years old) no structural abnormalities are found during autopsy.
- A more mandatory approach with systematic autopsy in sudden death cases leads to a very high percentage of explained causes of death.

## Introduction

Unexpected death in children and adolescents is an uncommon event with previously described incidences of 1,5 to 7,5 per 100.000 person-years, depending on the age categories included [1, 2]. Thorough investigation of the cause of death can be of great support for the grieving parents, and may have implications for genetic counselling in case of unexpected hereditary disease [3].

Most studies regarding the cause of death in children focus on sudden infant death syndrome (SIDS) in the very young (<1 year). In larger age cohorts, comprising children, adolescents and young adults, cardiovascular diseases seem to be an important cause of sudden unexpected death. In these studies arrhythmogenic disorders, coronary artery disease, myocarditis and cardiomyopathies were important contributors [4–6]. However, the results of these studies are dominated by the observations amongst young adults. Until thus far, few studies focused on the complete group of unexplained and unexpected deceased minors (<18 years), especially not in an autopsy-based and population-wide continuous cohort.

During a 15-month period a nationwide project regarding sudden death in children and adolescents (0–17 years) was carried out in the Netherlands [7]. This project was initiated by the government with the aim to gain insight in potentially hidden child abuse with fatal outcome among all minors in the Dutch population. As a valuable spin-off, this project allowed us to get information on the nationwide prevalence and causes of natural death due to cardiovascular disease, which is considered to be crucial for further cardiogenetic screening of relatives of the deceased [6].

In this autopsy study, we report the type and extent of cardiovascular diseases in unexplained and unexpected deaths in the underage population (0–17 years) of the Netherlands. Furthermore, we evaluated to what extent cardiovascular findings at autopsy could potentially have implications for cardiogenetic screening of relatives of the deceased children.

## Material and methods

In the Netherlands, over a period of 15 months (October 2012–December 2013), a detailed multidisciplinary investigation was required by law for all cases of unexplained and unexpected death in minors (0–17 years). This multidisciplinary investigation, the NODO procedure (*nader onderzoek doodsoorzaak*—closer investigation into cause of death), consisted of full medical and medico-social history, external examination, radiologic examination, laboratory examination and a medical autopsy. Consent from the parents was needed for the autopsy. The investigation was followed by a final expert consensus meeting in order to determine the manner and cause of death in each case [7]. In case of suspicion of a non-natural cause of death with the possibility of a crime, the public prosecutor seized the body for forensic autopsy. Because the findings of forensic autopsy are confidential, we only report on the findings of the medical autopsies.

All radiology and autopsy reports, as well as all summaries filled in for the expert consensus meetings were collected for all cases included in the procedure. The cases in whom an autopsy was not performed were excluded. A body autopsy with or without cerebral autopsy was performed following an extensive standardised protocol by specially trained pathologists. A detailed description of the cardiovascular examination is available in the online supplement.

## Results

**Fig. 1:**
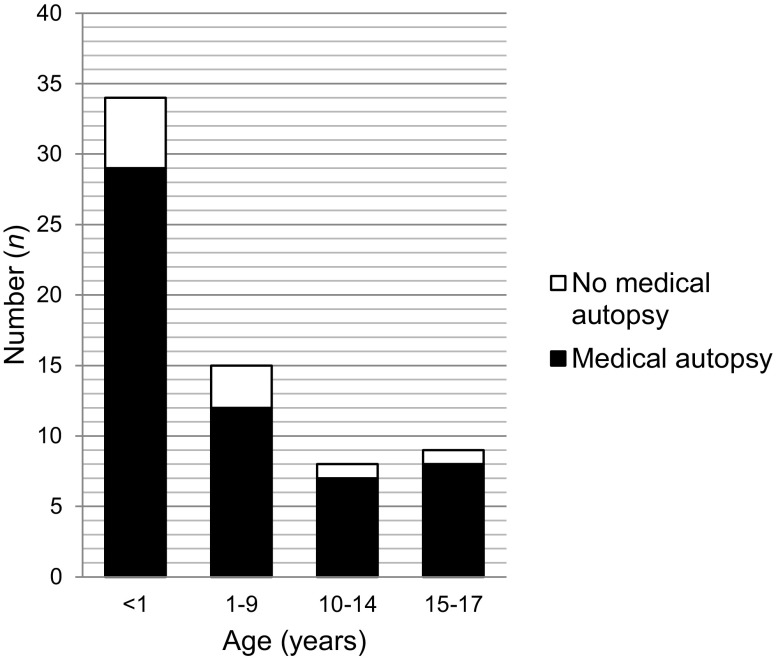
Cases included in the NODO procedure according to age

During the 15-month period, 66 cases from 11 of the 12 regions of the Netherlands were included in the NODO procedure. In two cases a forensic autopsy was performed and these cases were excluded from the analyses. In 56/66 cases (85%) a medical autopsy was performed. A complete autopsy was performed in 48 cases, a body autopsy without cerebral autopsy in 7 cases and a partial autopsy of heart and lungs in only 1 case. Ages of autopsied cases ranged from 2 days to 17 years and 11 months. Of these, 29 cases were below the age of 1. The other age categories included: 12 children aged 1–9 years, 7 children aged 10–14 years, and 8 adolescents aged 15–17 years (Fig. 1).

### Cardiovascular cause of death

In 42/56 cases (75%) of the autopsied cases a probable cause of death was found, with in 13/56 (23%) a cardiovascular cause of death (all causes of death are presented in the online supplementary Tables I and II). These cardiovascular causes of death were more frequently observed in the older age categories. A gender difference was not found.

**Fig. 2:**
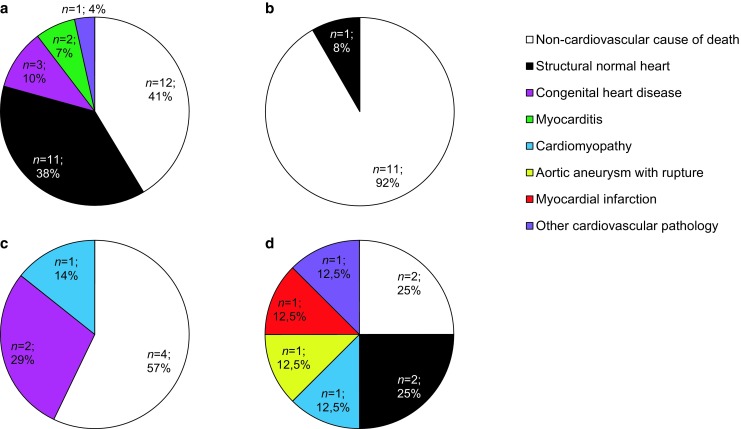
Cardiovascular causes of death in the different age categories. **a** Age <1; **b** Age 1–9, **c** Age 10–14, **d** Age 15–17

**Fig. 3:**
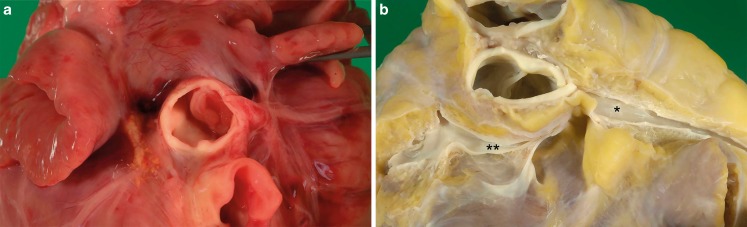
Congenital anomalies. Two examples of congenital abnormalities leading to death. In **a** congenital aortic stenosis due to narrowing of the valvar orifice by the thick valve cusps of a dysplastic valve. In **b** an anomaly of the main stem of the left coronary artery (**) arising from the right sinus next to the right coronary artery (*). View from posterior

Below the age of 1 a cardiovascular cause of death was found in 6/29 cases (21%; Fig. 2). These were mainly congenital abnormalities (*n* = 3) and fulminant myocarditis (*n* = 2). The congenital abnormalities were congenital aortic stenosis due to dysplastic aortic valve leaflets (Fig. 3), complete unbalanced atrioventricular septal defect, and an anomaly of the coronary arteries. The other cardiovascular cause of death was acute decompensated heart failure (with extensive ascites, pleural effusions and pulmonary siderophages) with unknown underlying cause.

In cases aged 1–9 years old, no cardiovascular causes of death were found.

In cases aged 10–14 years old, 3/7 cases (43%) suffered from a cardiovascular cause of death (Fig. 2). In two cases a congenital abnormality was found (67%). These were both anomalous origins of a coronary artery with histological features of myocardial ischaemia in the perfusion area of the affected artery (Fig. 3). In a 12-year-old male evident fibrofatty replacement was observed in the right ventricle wall, which fitted the diagnostic criteria of arrhythmogenic right ventricular cardiomyopathy (ARVC).

**Fig. 4:**
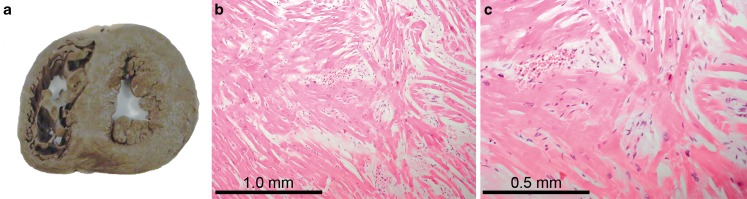
Cardiomyopathy. A hypertrophic cardiomyopathy as the cause of death in a 17 year old male. In **a** gross appearance of the heart with concentric hypertrophy. In **b** and **c** myocardial disarray, compatible with the diagnosis hypertrophic cardiomyopathy

In cases aged 15–17 year, a cardiovascular death was found in 4/8 cases (50%; Fig. 2). These causes were a hypertrophic cardiomyopathy with myocardial disarray (Fig. 4), an infectious endocarditis in a 15-year-old female with an artificial pulmonic valve, a ruptured aneurysm of the ascending aorta (due to severe medial degeneration) which resulted in hemopericardium, and a myocardial infarction due to unknown cause (no coronary artery pathology).

### Sudden death without an anatomical substrate

In 14/56 cases (25%) no structural abnormalities were found during autopsy and a cause of death could not be determined. A sudden death without anatomical substrate was mainly seen in patients <1 year of age (11/29 cases in this age group; 38%; Tab. 1). The cases were classified as sudden unexpected death in epilepsy (SUDEP, *n* = 2), sudden infant death syndrome (SIDS, *n* = 11) or sudden unexpected death syndrome (SUDS, *n* = 1) according to age and medical history.

**Table 1:** Patient characteristics

		All children with cardiovascular autopsy (*n* = 56)	Cardiovascular cause of death (*n* = 13)	No structural abnormalities (*n* = 14)
Age (years)	<1	29	6 (21%)	11 (38%)
	1–9	12	0 (0%)	1 (8%)
	10–14	7	3 (43%)	0 (0%)
	15–17	8	4 (50%)	2 (13%)
Gender	Male	31	8 (26%)	6 (19%)
	Female	25	5 (20%)	8 (32%)

### Cardiovascular findings of uncertain significance

Additionally, we found cardiovascular abnormalities of which the association with the death was uncertain [8]. These cardiovascular abnormalities of uncertain significance were present in 14/56 cases (25%). A more detailed description of these findings is available in the online supplement.

## Discussion

In this nationwide Dutch study on unexpected and unexplained death in minors (0–17 year) a series of 56 cases were autopsied. There were three important results: First, we found a cardiovascular cause of death in 23%. Second, in 25% no structural abnormalities were found, suggesting that further investigation for inherited arrhythmogenic disorders is indicated. Third, cardiac abnormalities ‘of uncertain significance’, that could not explain the death, were found in 25%.

The finding of a total of 48% of patients with a cardiovascular cause of death or an unexplained cause of death after thorough investigation is comparable with a previous study describing cardiovascular or unexplained deaths in 67/128 cases (52%) of unexpected sudden death aged 1–20 years in England [9]. Higher percentages were found in an Hispanic study in 34 sudden unexpected death cases aged 1–19 years old (62%) and a US study in 70 sudden unexpected death cases aged 0–20 years old (73%) [10, 11]. Possibly, the relatively low number of cases in the Hispanic study and the higher incidence of SIDS in the US can explain these differences [12]. A Dutch study also described a higher percentage of (presumed) cardiac cases in 115 patients with a natural cause of out-of-hospital cardiac arrest (78%). However, in this study autopsy results are lacking [13].

### Congenital anomalies

Congenital anomalies were found in 5/56 (9%) of autopsied cases, representing 39% (5/13) of all cases with a cardiovascular cause of death. Congenital anomalies were found to be the cause of death in the very young (<1 year) and between 10 and 14 years of age. The prevalence of 39% is comparable with the 41% congenital anomalies as a cardiovascular cause of death found in a recent large database study in the US [14]. In this study, also more deaths related to congenital heart disease were found in the younger patients and deaths associated with coronary artery anomalies occurred more often in the older categories (>10 years). Our observation that coronary anomalies are related to sudden death in the 10–15 year olds confirm the results of a study from Virmani et al. [15].

### Cardiomyopathies

Cardiomyopathies were found in 2/56 cases (4%). Paediatric cardiomyopathy is a rare disease with a reported annual incidence of 1,13 cases per 100.000 children in the US [16]. The finding of two cardiomyopathies in 13 cases of cardiovascular death (15%) is more or less comparable to the 12% found in the earlier described database study in the US, and to the 14% found in patients aged 1–20 years included in an Australian study regarding sudden cardiac death [14, 17].

### Myocarditis

In 2/56 cases (4%; 15% (2/13) of all cases with a cardiovascular cause of death), fulminant myocarditis was considered the cause of death. Both patients were <1 year old. In the earlier described database study in the US, myocarditis was found to be the cause of death in 5% of patients, most often aged 3–10 years old. In this study, an autopsy was only performed in 40% of cases [14]. In a study among young competitive athletes, myocarditis was found to be the cause of cardiovascular death in 6%. However, these patients were, although young, significantly older than our population [18]. A previous study in sudden unexpected deaths in children and adolescents (1–20 years) found myocarditis to be the cause of cardiovascular death in 16/53 (30%) cases [17].

### No structural abnormalities

In 14/56 cases (25%) no structural abnormalities were found, despite thorough investigation. The finding of no structural abnormalities can guide further (genetic) investigations in both the deceased and his relatives. Previous studies have shown that 27% of the sudden deaths with negative autopsy in young patients (1–35 years) are likely caused by hereditary arrhythmogenic disorders [17]. Furthermore, cardiogenetic screening of the relatives of young sudden death victims (1–18 years) without structural abnormalities during autopsy revealed a diagnosis in 11/22 cases [19]. The finding of inherited causes of sudden death can prevent future events in these families.

In our study, most (11/14) cases without structural abnormalities were sudden infant deaths (<1 year old). In a previous study in a sudden infant death cohort, genetic variants with likely functional effects in genes associated with channelopathies and cardiomyopathies were found in 34% of patients [20]. Based on these findings, it is likely that at least a number of our cases without major structural disease at autopsy suffered an arrhythmic death due to an inherited disease. Therefore, we suppose that the 23% of cases with a definite cardiovascular cause of death likely reflects an underestimation of the real burden of cardiovascular disease, and especially of inherited cardiovascular disease. Genetic testing in the different age categories in sudden death in children and adolescents is an interesting topic for future studies. Regrettably, genetic analysis was not available in most cases in our study cohort, since it was not included in the procedure and requires specific permission of relatives.

### Limitations

Because unexpected and unexplained death in children is a rare event, only a relatively low number of cases could be included in this study. Since we depended on registered cases, some cases could have been missed. Missing completely at random is expected in these cases and therefore these missing cases will not influence our results of relative frequencies of cardiovascular causes of death. In two cases a forensic autopsy was performed, of which the results are confidential and could not be included in the study. By excluding these cases with theoretically a lower risk on cardiovascular causes of death (due to the suspicion of a non-natural cause of death), there might be a slight selection bias.

### Future perspectives

During the NODO procedure, investigation into the cause of death of minors was regulated by law in the Netherlands and this period has now ended. Although in the follow-up of this procedure finance for further investigation of causes of death in children became available for a limited time period of three years, death cause investigation has not yet been structurally organised in the Netherlands. Health insurance stops after death, therefore structural finance for autopsies is lacking. In addition, the logistics of arranging an autopsy can be quite laborious for general practitioners and forensic physicians involved in sudden death cases. Therefore, the possibility and importance of a medical autopsy is not discussed with the family members in all cases in busy daily practice. In other countries, such as the UK, in sudden unexpected death cases autopsy is mandatory, which might be an instrument to increase autopsy rate. We think the NODO procedure, a procedure that elucidated the cause of sudden unexpected death in 75% of cases, has proven that this more mandatory approach indeed leads to a very high percentage of explained causes of sudden death.

In conclusion, this national cohort with high autopsy rate reveals a high incidence of macroscopically and/or microscopically visible cardiovascular diseases contributing to sudden unexpected death in children. In another high percentage of victims with only minor or no structural abnormalities a substantial number of genetic arrhythmias as cause of death may be expected. These findings underline the importance of a systematic autopsy in sudden death in the young with implications for cardiogenetic screening of relatives.

## Caption Electronic Supplementary Material

The Electronic Supplementary Material provides additional information about the autopsy procedure, the causes of death, and the cardiovascular findings of unknown significance.
