# Correction to: Cardiovascular causes of sudden unexpected death in children and adolescents (0–17 years): A nationwide autopsy study in the Netherlands

**DOI:** 10.1007/s12471-018-1219-9

**Published:** 2019-01-08

**Authors:** A. Vos, A. C. van der Wal, A. H. Teeuw, J. Bras, A. Vink, P. G. J. Nikkels, Dutch NODO group

**Affiliations:** 1 Department of Pathology, University Medical Center, Utrecht, The Netherlands; 2 Department of Pathology, Academic Medical Center, Amsterdam, The Netherlands; 3 Department of Paediatrics, Academic Medical Center, Amsterdam, The Netherlands

**Correction to:**

**Neth Heart J 2018**

10.1007/s12471-018-1152-y

In the version of the article originally published online, there was an error in the ‘Methods and results’ section of the Abstract. It is stated that ‘In the 10–14 year group, hypertrophic cardiomyopathy (*n* = 1) and ruptured ascending aortic aneurysm (*n* = 1) were among the observed cardiovascular causes.’

The line should have read: ‘In the 10–14 year group, coronary anomalies (*n* = 2) and arrhythmogenic cardiomyopathy (*n* = 1) were observed. In the 15–17 year group, hypertrophic cardiomyopathy (*n* = 1) and ruptured ascending aortic aneurysm (*n* = 1) were among the observed cardiovascular causes.’

