# Supplementary material for: Cardiovascular causes of sudden unexpected death in children and adolescents (0–17 years): A nationwide autopsy study in the Netherlands
Source: Neth Heart J. 2018 Sep 3;26(10):500–5. doi: 10.1007/s12471-018-1152-y (PMC6150875; doi:10.1007/s12471-018-1152-y)
Supplement: Supplementary file 1 — The Electronic Supplementary Material provides additional information about the autopsy procedure, the causes of death, and the cardiovascular findings of unknown significance. [file 12471_2018_1152_MOESM1_ESM.pdf]

Online data supplement

This data supplement has been provided by the authors to give the readers additional information about their work.

Supplement to: Vos A, van der Wal AC, Teeuw AH, Bras J, Vink A, Nikkels PGJ. Cardiovascular causes of sudden unexpected death in children and adolescents (0-17 years); a nationwide autopsy study in the Nederlands.

Table of contents

Content	Page
Supplemental methods: Cardiovascular autopsy	2
Supplemental results: Cardiovascular findings of uncertain significance	3
Supplemental discussion: Borderline myocarditis	5
Supplemental figure I: Borderline myocarditis	6
Supplemental table I: Cardiovascular causes of death and structural normal hearts	7
Supplemental table II: Non-cardiovascular causes of death	8
References	9

Supplemental methods

Cardiovascular autopsy

Examination of the heart and great arteries included recording of heart weight, developmental abnormalities, measurement of the atrial and ventricular dimensions, myocardial thickness, structure and insertion of cardiac valves and aortic root including coronary ostia. Macro-enzyme (LDH) staining of one entire biventricular myocardial slice was perfomed for detection of acute cardiomyocyte damage. Sections for histologic examinations were taken from both ventricles (in most cases a complete biventricular heart slice) and atrioventricular nodal area/interventricular septum and stained with haematoxylin and eosin (H&E).

If a myocarditis was suspected on routine H&E stained slides, additional CD3 and CD68 immunohistochemical stains were performed to detect T-lymphocytes and macrophages respectively. In cases of presence of focal infiltrates with only discrete myocardial damage, the case was categorized as borderline myocarditis.[1] Diffuse inflammatory infiltration with substantial myocardial damage was categorized as fulminant myocarditis.

Supplemental results

Cardiovascular findings of uncertain significance

In 13/56 autopsied cases (23%) a cardiovascular cause of death was found. Additionally, we found cardiovascular abnormalities of which the association with the death was uncertain.[2] These cardiovascular abnormalities of uncertain significance were present in 14 cases (25%). In one of these cases another cardiovascular disease was found to be the main cause of death. In 4 cases no other cause of death was found and the cases were classified as 'no structural abnormalities'. In total, cardiovascular abnormalities (cardiovascular causes of death and cardiovascular findings of uncertain significance) were found in 26/56 cases. (46%)

Findings of uncertain significance included: small atrium septum defect (n=2), abberant right subclavian artery/arteria lusoria (right subclavian artery originating from the descending aorta and running behind the esophagus to the right (n=2)), pulmonary venolobar (Scimitar) syndrome which was already diagnosed before death (n=1), abnormal outlet of the coronary sinus in the left atrium (n=1), a high take off of the left coronary artery with a collateral artery connecting the right coronary artery to the left without histological signs of acute ischemia (n=1), and borderline/focal myocarditis (n=8).

Borderline myocarditis

In 2/56 (4%) cases a fulminant myocarditis was found to be the cause of death. In one of these cases a parvovirus was detected in the myocardium. In another 8/56 cases (14%), age range 1 to 14 years, one or more clustered myocardial infiltrates of T-lymphocytes and macrophages, with focal indication of cardiomyocyt damage, were present (online supplemental figure I). These were diagnosed as borderline lymphocytic myocarditis according to the Dallas criteria.[1] Histologic examination of the remaining organs showed similar infiltrates in

larynx, trachea, lungs, tonsils, liver and/or brain in 7 of 8 cases. A cause of death, unrelated to the focal myocarditis, was found in 5 of these cases (intussusception with pulmonary edema, gangrenous purpura, brain herniation due to dysfunction of a ventricular-peritoneal shunt, and pneumonia (2x)). The other three children died of unknown causes, since borderline myocarditis is considered to be an uncertain cause of death.[2]

Supplemental discussion

Borderline myocarditis

We found limited focal lymphocytic myocarditis in 8/56 cases (14%), which, according to the recent guidelines of autopsy in sudden cardiac death, should be interpreted as at most an uncertain cause of death.[2] Our results, with an evident cause of death other than the borderline myocarditis in 5/8 cases, confirm this statement. Since we also found mild lymphohistiocytic infiltrates in the liver, lungs and/or brain in most of these cases (7 out of 8), this focal myocarditis most likely represents a generalized viral infection, as suggested by Bajanowski et al.[3] Unfortunately, systematic viral cultures or polymerase chain reactions of the myocardium to confirm this statement are lacking. Our results endorse the view to be cautious with interpreting a focal myocarditis as the cause of death. Especially since erroneous interpretation of insignificant findings at autopsy may masquerade the presence of an unnoticed inherited arrhythmogenic disease.[4]

Supplemental figure I: Borderline myocarditis

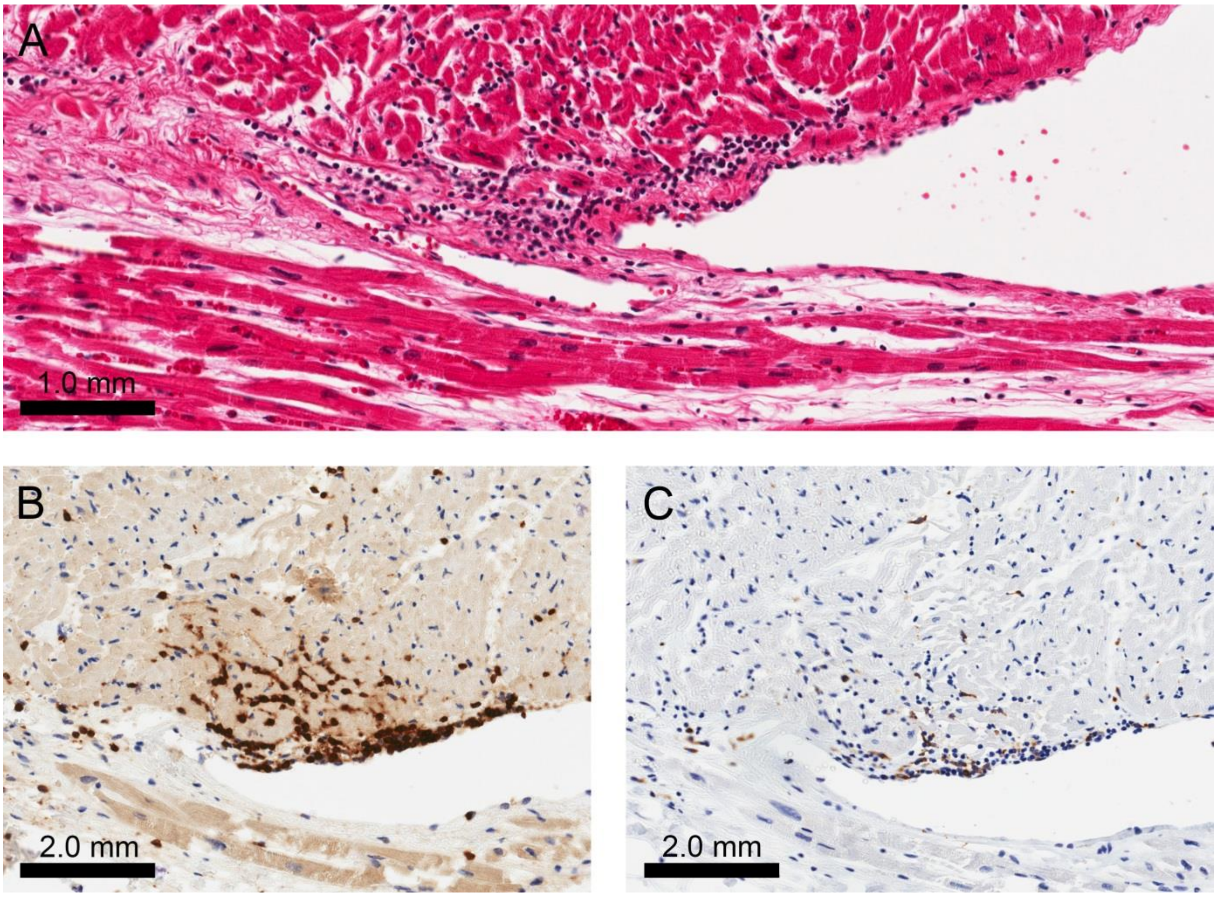

An example of a focus of lymphohistiocytic infiltrate with focal cardiomyocyte damage on H&E staining (A), with immunohistochemical analysis of the infiltrate. (CD3 positive T-lymphocytes in B and CD68 positive macrophages in C)

Supplemental table I: Cardiovascular causes of death and structural normal hearts

Cardiovascular causes of death

	Age	*Sex*	*Cause of death*	*Circumstances of death*
1	4 d	ma	Congenital aortic stenosis	During sleep
2	5 d	ma	Complete atrioventricular septal defect	During sleep
3	8 d	fe	Sudden Infant Death Syndrome	During sleep
4	1 m	fe	Sudden Infant Death Syndrome	During sleep
5	1.5 m	ma	Acute decompensated heart failure	During bathing
6	1.5 m	fe	Fulminant myocarditis	During feeding
7	1,5 m	fe	Sudden Infant Death Syndrome	During sleep
8	2 m	ma	Sudden Infant Death Syndrome	During sleep
9	2.5 m	fe	Sudden Infant Death Syndrome	During sleep
10	3 m	ma	Sudden Infant Death Syndrome	During sleep
11	3 m	fe	Anomalous origin left coronary artery	During feeding
12	3.5 m	ma	Sudden Infant Death Syndrome	During sleep
13	5 m	ma	Fulminant myocarditis	During transportation to emergency unit
14	5.5 m	fe	Sudden Infant Death Syndrome	During sleep
15	7 m	ma	Sudden Infant Death Syndrome	During sleep
16	8 m	fe	Sudden Infant Death Syndrome	During sleep
17	8 m	ma	Sudden Infant Death Syndrome	During sleep
18	3.3 y	ma	Sudden Unexpected Death Syndrome	During sleep
19	10.1 y	ma	Anomalous origin left coronary artery	During exercise
20	12.3 y	ma	Arrhythmogenic right ventricular cardiomyopathy	At home
21	13.7 y	ma	Anomalous origin left coronary artery	During exercise
22	15 y	fe	Sudden Unexpected Death in Epilepsy	During sleep
23	15.6 y	ma	Sudden Unexpected Death in Epilepsy	During sleep
24	15.7 y	fe	Infectious endocarditis (of artificial pulmonic valve)	During transportation to emergency unit
25	17.2 y	fe	Ruptured aneurysm of ascending aorta with hemopericardium	Found death next to the private pool
26	17.8 y	ma	Hypertrophic cardiomyopathy with myocardial disarray	Drowned
27	17.9 y	fe	Myocardial infarction due to unknown cause	Found death next to her bike

d=days, m=months, y=years, ma=male fe=female

Supplemental table II: Non-cardiovascular causes of death

Non-cardiovascular causes of death

	Age	*Sex*	*Cause of death*	*Circumstances of death*
1	2 d	fe	Pulmonary hypoplasia	At home
2	18 d	ma	Cerebral hypoxia	During sleep
3	1.5 m	ma	Urosepsis	At home
4	2 m	ma	Cerebral hypoxia	During sleep
5	2 m	ma	Pneumonia	During sleep
6	3.5 m	fe	Pneumonia	During sleep
7	3.5 m	ma	Waterhouse Friderichsen syndrome	At home
8	4 m	fe	Pneumonia	During sleep
9	4.5 m	fe	Pneumonia	During sleep
10	5 m	ma	Pneumonia	During sleep
11	10 m	fe	Metabolic disorder	During sleep
12	11.9m	ma	Hypovolemic shock due to purpura fulminans	At home
13	12.5 m	ma	Strangulation of the bowel with ischemia	At home
14	14 m	ma	Pneumonia	During sleep
15	14 m	ma	Pneumonia and acute gastroenteritis with hypovolemia	At home
16	14 m	fe	Pneumonia	In the hospital
17	15.5 m	ma	Severe hypovolemia due to acute tubular damage	During sleep
18	16 m	fe	Pneumonia	During sleep
19	2 y	fe	Strangulation of the bowel with ischemia	During sleep
20	4.3 y	ma	Pneumonia	During sleep
21	4.3 y	ma	Intussusception due to Meckel's diverticulum	At home
22	6.9 y	fe	Strangulation of the bowel with ischemia	During sleep
23	9.6 y	ma	Brain herniation due to ventriculoperitoneal shunt dysfunction	During sleep
24	10.5 y	ma	Pneumonia	In the hospital
25	11.9 y	ma	Diabetic ketoacidosis	During sleep
26	12.7 y	fe	Pneumonia	During sleep
27	13.4 y	ma	Pulmonary hemorrhage	During sleep
28	15.2 y	ma	Thyrotoxicosis	During sleep
29	17.8 y	fe	Acute cerebral edema with aqueduct anomaly	During sleep

d=days, m=months, y=years, ma=male fe=female

References

1. Aretz HT. Myocarditis: the Dallas criteria. Human pathology 1987;18:619-24.
2. Basso C, Aguilera B, Banner J et al. Guidelines for autopsy investigation of sudden cardiac death: 2017 update from the Association for European Cardiovascular Pathology. Virchows Archiv : an international journal of pathology 2017;471:691-705.
3. Bajanowski T, Ortmann C, Teige K et al. Pathological changes of the heart in sudden infant death. International journal of legal medicine 2003;117:193-203.
4. Papadakis M, Raju H, Behr ER et al. Sudden cardiac death with autopsy findings of uncertain significance: potential for erroneous interpretation. Circulation Arrhythmia and electrophysiology 2013;6:588-96.
